# Supplementary material for: Reducing methylation of histone 3.3 lysine 4 in the medial ganglionic eminence and hypothalamus recapitulates neurodevelopmental disorder phenotypes
Source: bioRxiv. 2025 May 2:2025.05.02.651761. Preprint. [Version 1] doi: 10.1101/2025.05.02.651761 (PMC12190312; doi:10.1101/2025.05.02.651761)
Supplement: 1 [file NIHPP2025.05.02.651761V1-supplement-1.pdf]

bioRxiv preprint doi: https://doi.org/10.1101/2025.05.02.651761; this version posted May 2, 2025. The copyright holder for this preprint (which was not certified by peer review) is the author/funder. This article is a US Government work. It is not subject to copyright under 17 USC 105 and is also made available for use under a CC0 license.

Figure S1

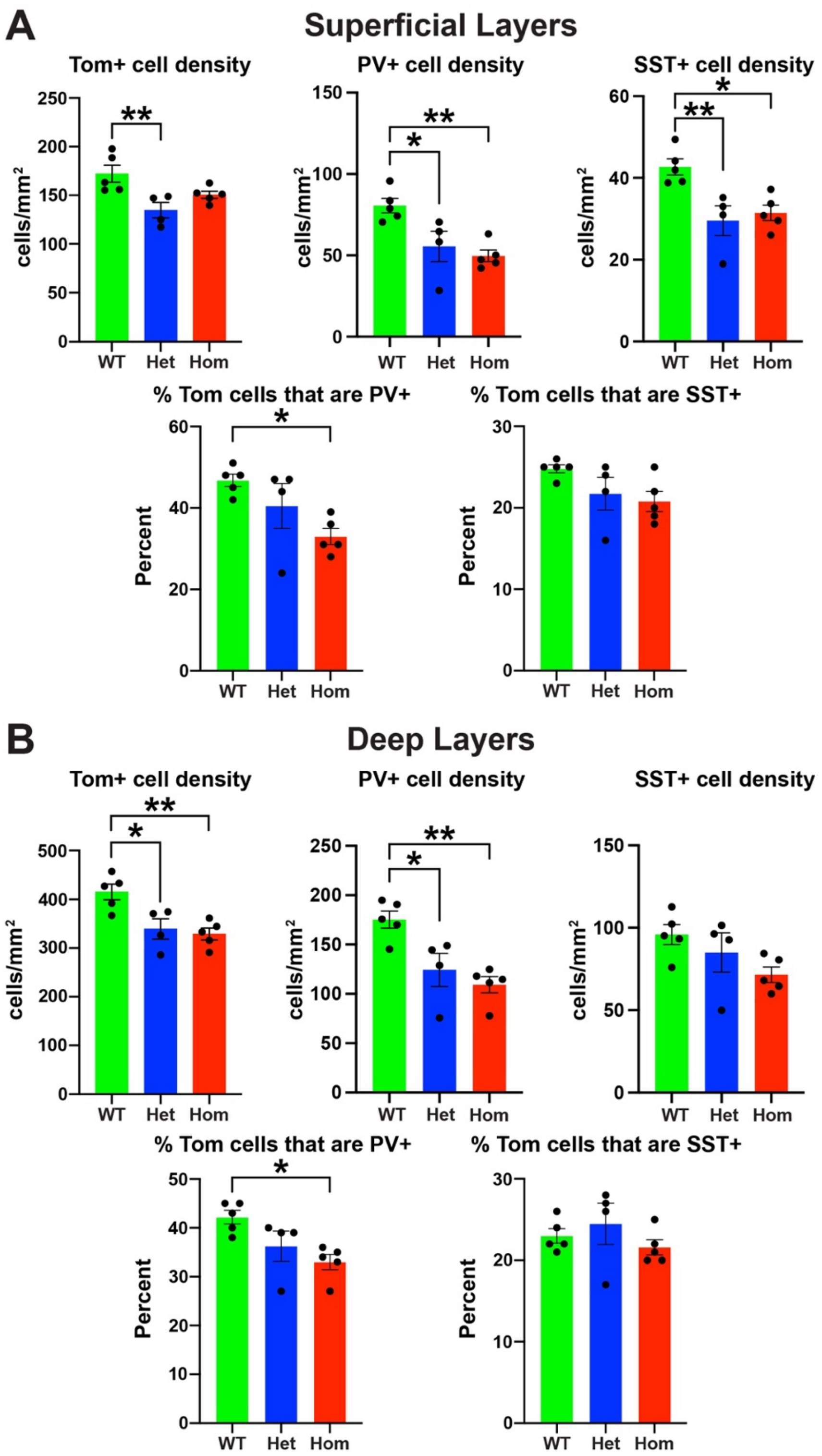

Figure S1. Decreased MGE-derived interneurons in the superficial and deep layers of H3.3K4M Hom mice cortex. A. Graphs depicting the density of Tom+, PV+ and SST+ cells (top) and the percent of Tom+ cells expressing PV or SST (bottom) in the superficial layers (I-III) of P21 *Nkx2.1-Cre*;*H3.3K4M*;*Ai9* mice. **B.** Graphs depicting the density of Tom+, PV+ and SST+ cells (top) and the percent of Tom+ cells expressing PV or SST (bottom) in the deep layers (IV-VI) of P21 *Nkx2.1-Cre*;*H3.3K4M*;*Ai9* mice. All stats are one-way ANOVA followed by Tukey's multiple comparison tests (A, B): * = p $\leq$ .05, ** = p $\leq$ .005.

bioRxiv preprint doi: https://doi.org/10.1101/2025.05.02.651761; this version posted May 2, 2025. The copyright holder for this preprint (which was not certified by peer review) is the author/funder. This article is a US Government work. It is not subject to copyright under 17 USC 105 and is also made available for use under a CC0 license.

Figure S2

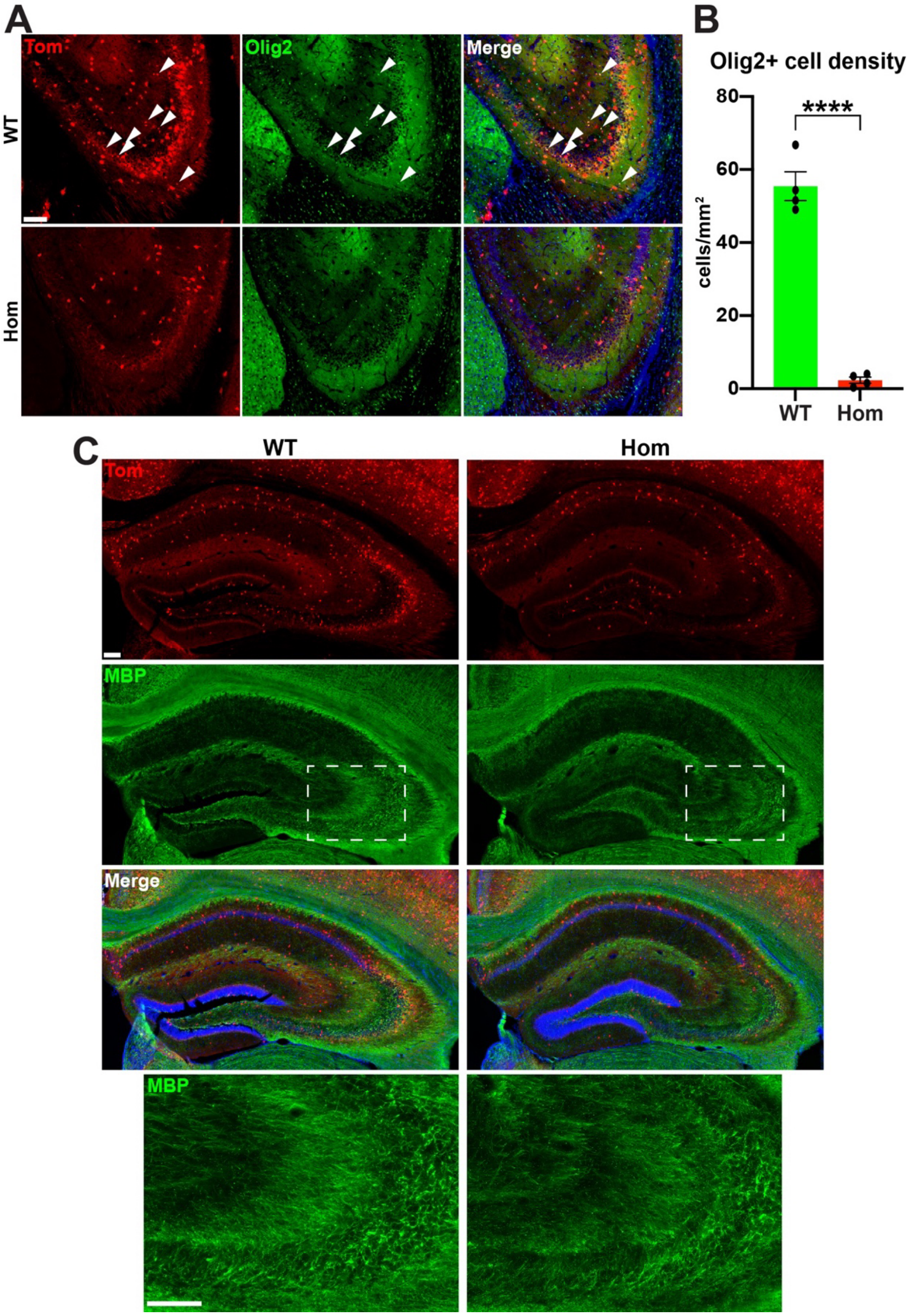

Figure S2. Loss of MGE-derived oligodendrocytes in H3.3K4M Hom mice. A. Representative images stained for Olig2 (green) through the CA3 showing loss of small cell body Tom+/Olig2+ MGE-derived oligodendrocytes (white arrowheads) in H3.3K4M Hom mice. **B.** Quantification of Tom+/Olig2+ oligodendrocytes in CA3 region of WT and Hom mice. Standard 2-tailed t-tests: ****$p \leq 0.0001$. **C.** Representative image showing reduction of myelin basic protein (MBP) in CA3 of H3.3K4M Hom mice. Scale bars = 50 μm.

bioRxiv preprint doi: https://doi.org/10.1101/2025.05.02.651761; this version posted May 2, 2025. The copyright holder for this preprint (which was not certified by peer review) is the author/funder. This article is a US Government work. It is not subject to copyright under 17 USC 105 and is also made available for use under a CC0 license.

Figure S3

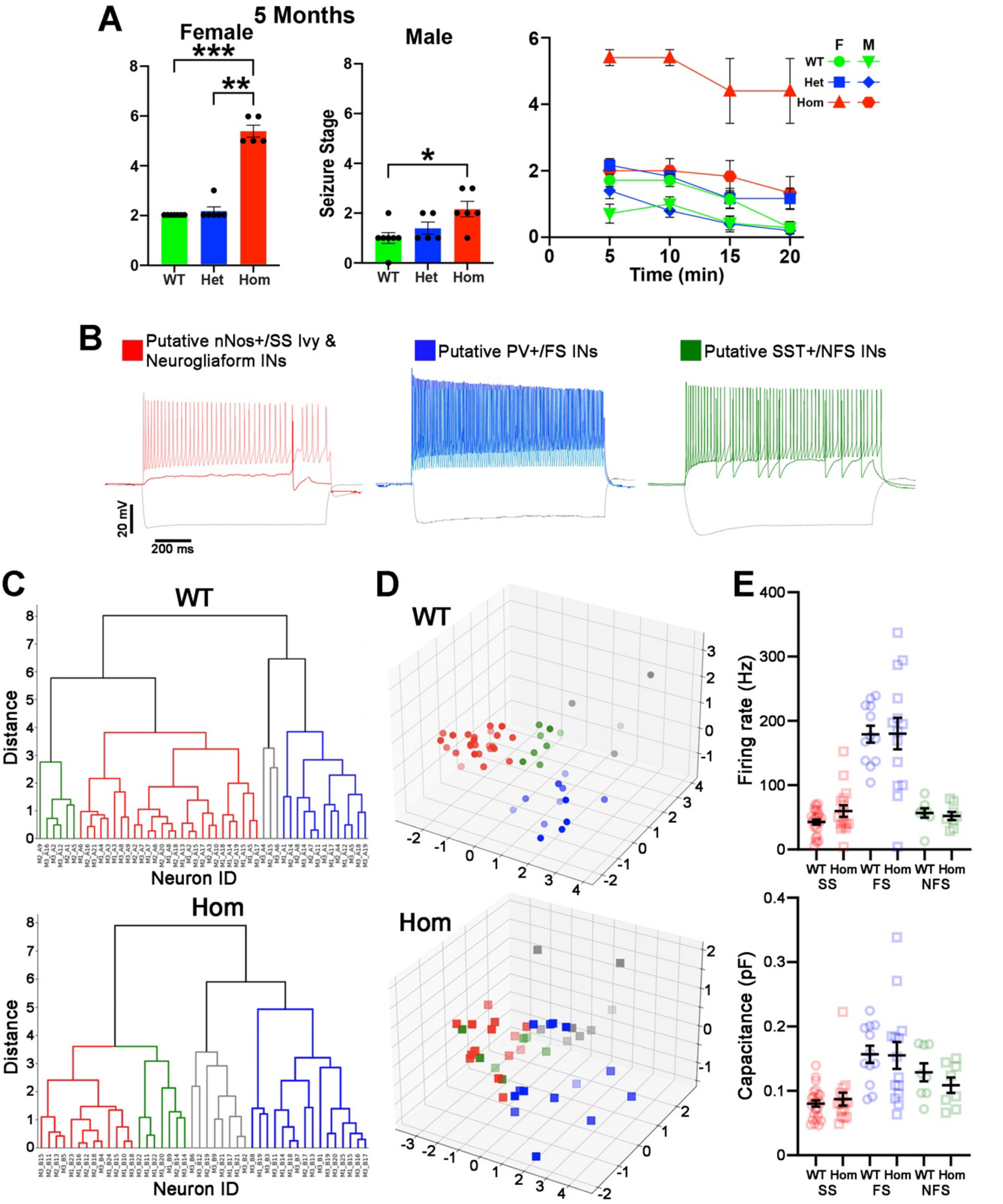

bioRxiv preprint doi: https://doi.org/10.1101/2025.05.02.651761; this version posted May 2, 2025. The copyright holder for this preprint (which was not certified by peer review) is the author/funder. This article is a US Government work. It is not subject to copyright under 17 USC 105 and is also made available for use under a CC0 license.

Figure S3. Altered intrinsic properties and network connectivity of hippocampal interneurons in H3.3K4M Hom mice. A. Maximum scores (seizure stage, left) for males and females following PTZ injection (20 mg/kg) at 5 months, and along the 20-minute observation period (right). **B.** Representative patch-clamp recording traces form putative nNos+/slow-spiking (SS, red), PV+/Fast Spiking (FS, blue) and SST+/non-FS (NFS, green) interneurons. **C-D.** Unbiased hierarchical clustering dendrograms (C) and PCA plots (D) of all hippocampal interneurons recorded from H3.3K4M WT (n = 49 cells) and Hom (n = 44 cells) mice. Cells that could not be classified are gray. **E.** Graphs depicting firing rate (top) and capacitance (bottom) for SS (red), FS (blue) and NFS (green) cells. Significant increase in variance in the FS population evaluated by F test in membrane capacitance (p = *) and firing rate (p = *) is observed in H3.3K4M Hom mice compared to WT. Kruskal-Wallis followed by Dunn's multiple comparisons (A: left, middle) or two-way ANOVA followed by Tukey's multiple comparison tests (A: right) were performed: * = $p \leq .05$, ** = $p \leq .005$, *** = $p \leq .0005$.

bioRxiv preprint doi: https://doi.org/10.1101/2025.05.02.651761; this version posted May 2, 2025. The copyright holder for this preprint (which was not certified by peer review) is the author/funder. This article is a US Government work. It is not subject to copyright under 17 USC 105 and is also made available for use under a CC0 license.

Figure S4

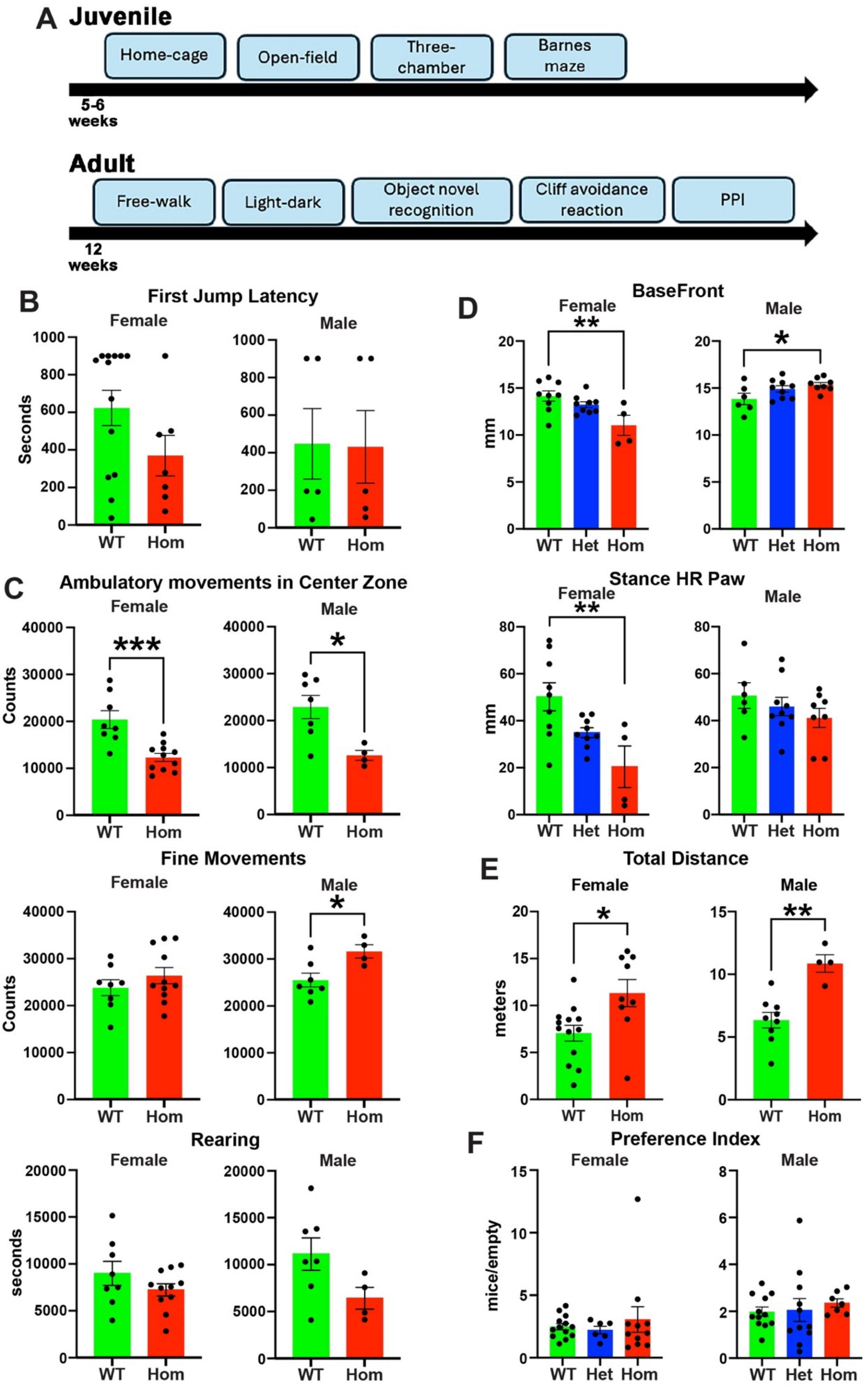

bioRxiv preprint doi: https://doi.org/10.1101/2025.05.02.651761; this version posted May 2, 2025. The copyright holder for this preprint (which was not certified by peer review) is the author/funder. This article is a US Government work. It is not subject to copyright under 17 USC 105 and is also made available for use under a CC0 license.

Figure S4. Increased anxiety and impaired locomotion in H3.3K4M Hom mice. A. Summary of behavior assays performed in juvenile and adult mice. **B.** First jump latency in the Cliff Avoidance Reaction Test. **C.** Beam break counts of ambulatory movements in the center zone (top), fine movements in the whole zone (middle) and rearing movements (bottom) over 4 days in the home cage test. **D.** BaseFront (top) and stance of hind right paw (bottom) in the free walk test. **E.** Total distance traveled by mice in the Barnes maze test. **F.** Preference index in the three-chamber test. All stats are one-way ANOVA followed by Tukey's multiple comparison tests when WT, Het and Hom mice (D, F); standard 2-tailed t-test when only WT and Hom mice tested (B, C, E): * = $p \leq .05$, ** = $p \leq .005$, *** = $p \leq .0005$.

bioRxiv preprint doi: https://doi.org/10.1101/2025.05.02.651761; this version posted May 2, 2025. The copyright holder for this preprint (which was not certified by peer review) is the author/funder. This article is a US Government work. It is not subject to copyright under 17 USC 105 and is also made available for use under a CC0 license.

Figure S5

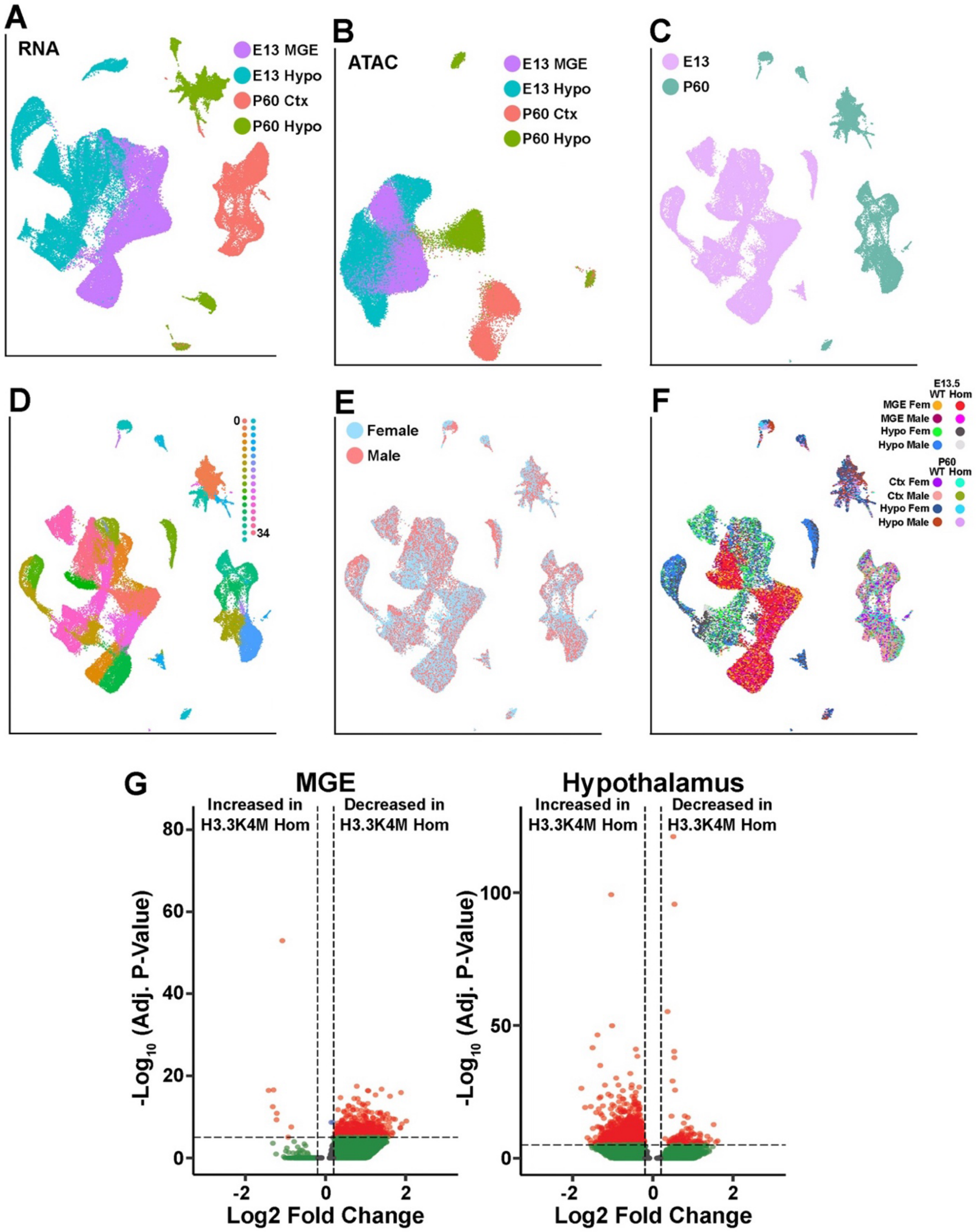

Figure S5. Total integrated Multiome-seq (snRNA-seq and snATAC-seq) data of 16 samples. A-B. UMAP plots of E13.5 MGE, E13.5 hypothalamus, P60 cortex and P60 hypothalamus annotated by age and tissue of the RNA-only (A) and ATAC-only (B). **C-F.** Integrated RNA and ATAC UMAP plots of E13.5 MGE, E13.5 hypothalamus, P60 cortex and P60 hypothalamus annotated by age (C), putative cell clusters (D), sex (E) and library id (F). **G.** Volcano plots depicting differentially accessible peaks from snATAC data in embryonic MGE (left) and hypothalamus (right) with combined sex.

bioRxiv preprint doi: https://doi.org/10.1101/2025.05.02.651761; this version posted May 2, 2025. The copyright holder for this preprint (which was not certified by peer review) is the author/funder. This article is a US Government work. It is not subject to copyright under 17 USC 105 and is also made available for use under a CC0 license.

Figure S6

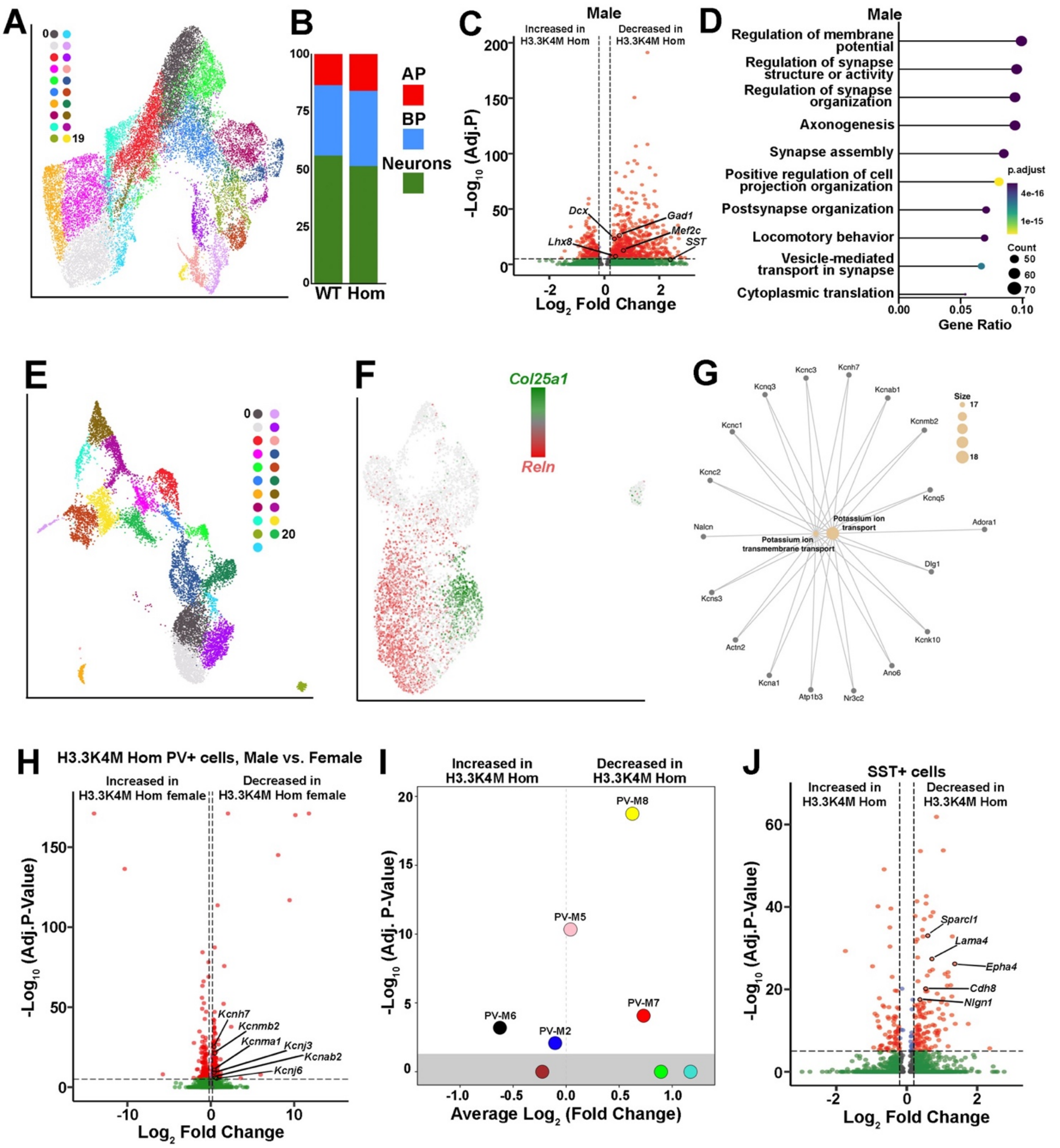

bioRxiv preprint doi: https://doi.org/10.1101/2025.05.02.651761; this version posted May 2, 2025. The copyright holder for this preprint (which was not certified by peer review) is the author/funder. This article is a US Government work. It is not subject to copyright under 17 USC 105 and is also made available for use under a CC0 license.

Figure S6. Altered transcriptome in MGE-derived interneurons associated with cell fate and seizure. A. UMAP plots of E13.5 MGE integrated single nuclei RNA- and ATAC-seq annotated by Seurat clusters. **B.** Proportions of E13.5 apical progenitors (AP), basal progenitors (BP) and neurons. **C.** Volcano plot depicting DEGs of male E13.5 MGE between H3.3K4M WT and Hom mice. **D.** clusterProfiler GO enrichment top biological processes for DEGs of male E13.5 MGE between H3.3K4M WT and Hom mice. **E.** Integrated UMAP plots of MGE-derived interneurons in P60 cortex annotated by Seurat clusters. **F.** UMAP plot of PV+ interneurons depicting *Reln* and *Col25a1* expression. **G.** Category netplot visualizing the GO terms of potassium ion transport and potassium ion transmembrane transport. **H.** Volcano plot depicting DEGs of P60 H3.3K4M Hom PV+ interneurons between female and male. **I.** Volcano plot depicting module eigengene differences identified by co-expression analysis in PV+ population between H3.3K4M WT and Hom mice. **J.** Volcano plot depicting DEGs of P60 SST+ interneurons between H3.3K4M WT and Hom mice.

bioRxiv preprint doi: https://doi.org/10.1101/2025.05.02.651761; this version posted May 2, 2025. The copyright holder for this preprint (which was not certified by peer review) is the author/funder. This article is a US Government work. It is not subject to copyright under 17 USC 105 and is also made available for use under a CC0 license.

Figure S7

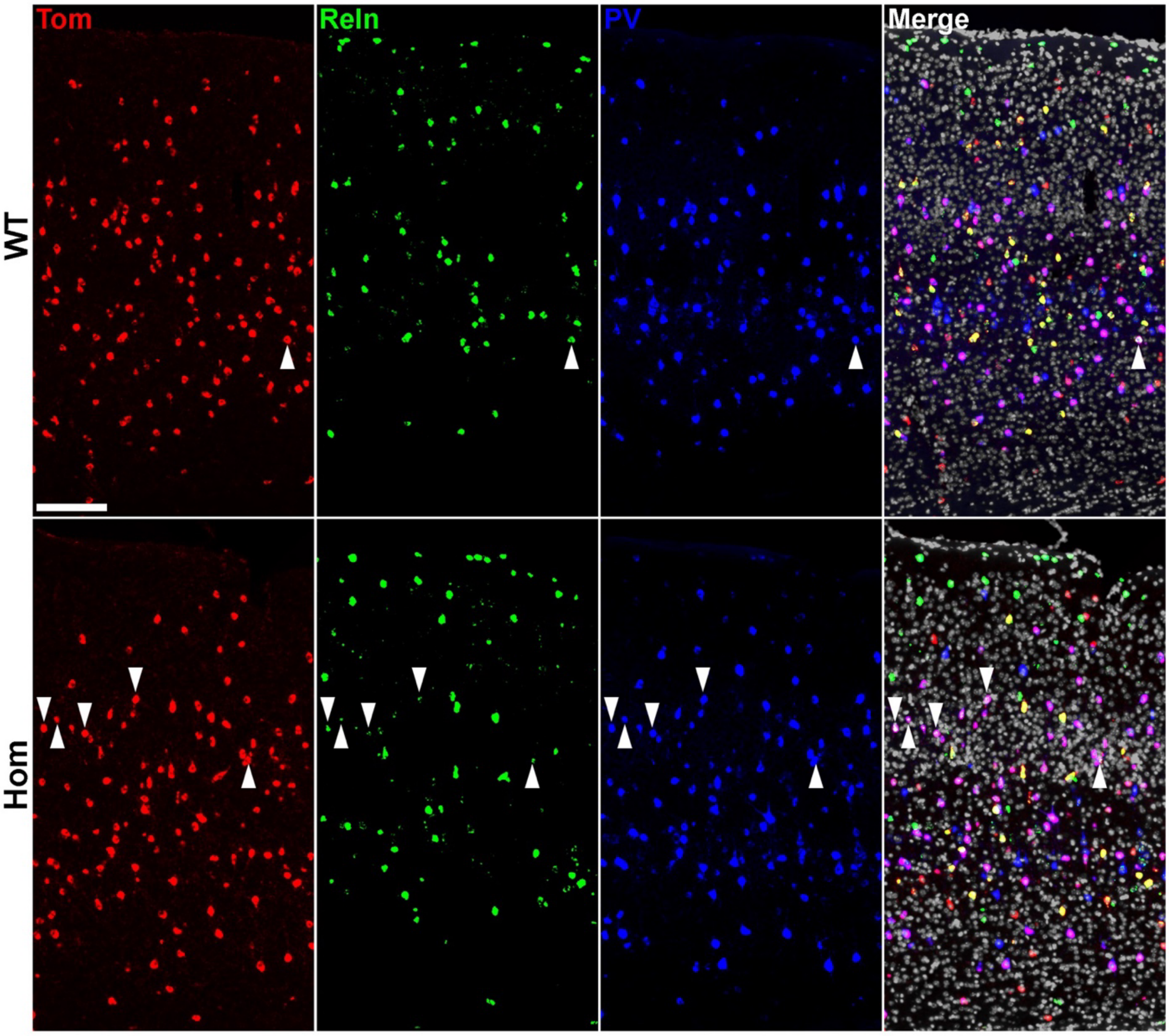

Figure S7. Increased PV+ / Reln+ in H3.3K4M Hom mice. Representative images of adult cortex showing increased number of Tom+/PV+/Reln+ cortical interneurons (white arrowheads) in H3.3K4M Hom mice compared to WT. Scale bar = 100 μm.

bioRxiv preprint doi: https://doi.org/10.1101/2025.05.02.651761; this version posted May 2, 2025. The copyright holder for this preprint (which was not certified by peer review) is the author/funder. This article is a US Government work. It is not subject to copyright under 17 USC 105 and is also made available for use under a CC0 license.

Figure S8

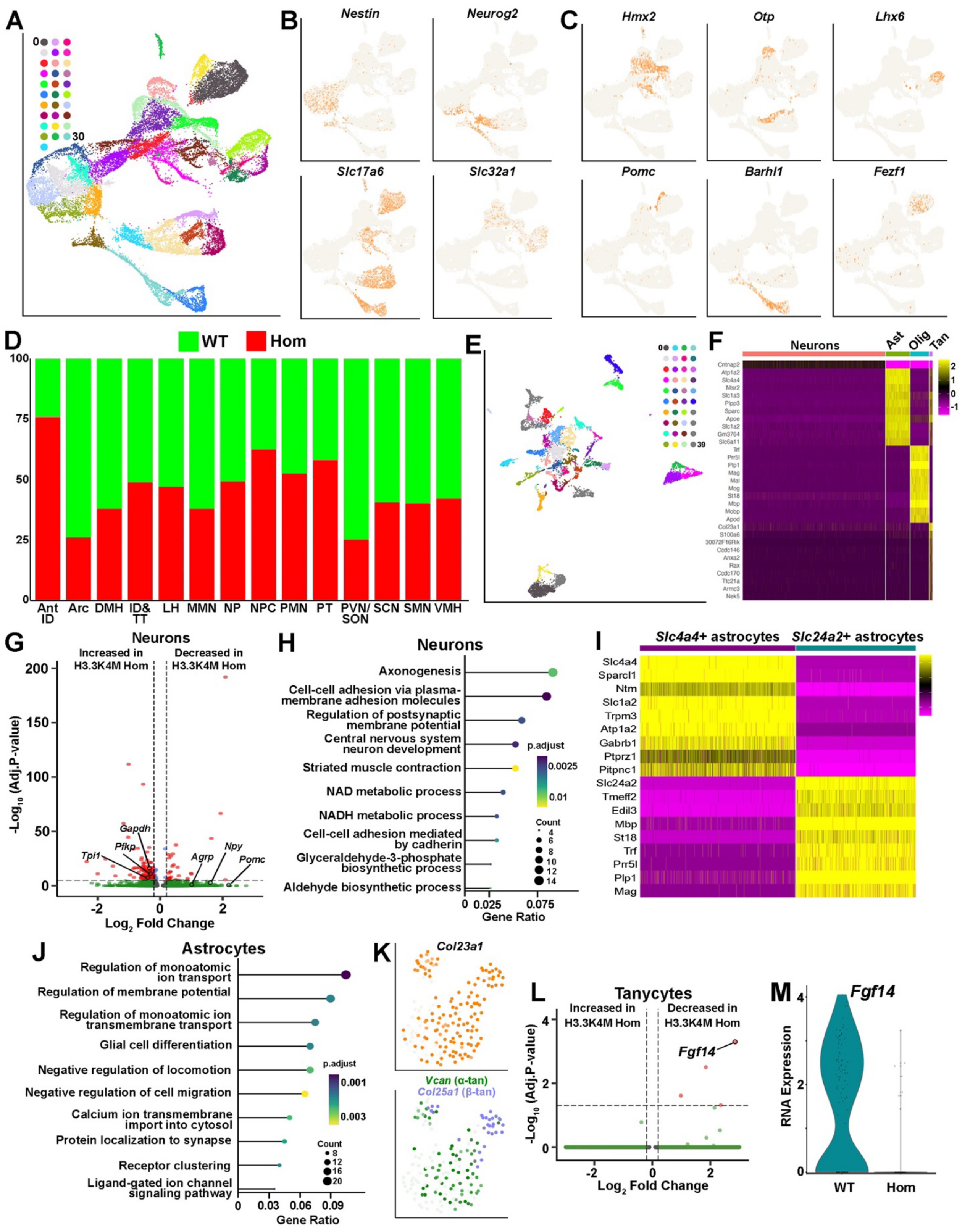

bioRxiv preprint doi: https://doi.org/10.1101/2025.05.02.651761; this version posted May 2, 2025. The copyright holder for this preprint (which was not certified by peer review) is the author/funder. This article is a US Government work. It is not subject to copyright under 17 USC 105 and is also made available for use under a CC0 license.

Figure S8. Transcriptome changes in numerous hypothalamic cell types in H3.3K4M Hom mice. A. Integrated UMAP plots of E13.5 hypothalamus annotated by Seurat clusters. **B.** Markers for apical progenitors (*Nestin*), neuronal precursors (*Neurog2*), postmitotic glutamatergic neurons (*Slc17a6*) and postmitotic GABAergic neurons (*Slc32a1*). **C.** Markers of region-specific hypothalamic nuclei PMN (*Hmx2*), DMH and PVN/SON (*Otp*), ID & TT (*Lhx6*), ARC (*Pomc*), SMN (*Barhl1*) and VMH (*Fezf1*). **D.** Relative proportions of hypothalamic nuclei/regions between H3.3K4M WT and Hom mice. **E.** Integrated RNA and ATAC UMAP plot of P60 hypothalamus annotated by Seurat clusters. **F.** Heatmap depicting critical genes that define astrocytes, oligodendrocytes and tanycytes. **G-H.** Volcano plot depicting DEGs of P60 hypothalamic astrocytes (G) and clusterProfiler GO enrichment of biological process for DEGs of P60 hypothalamic astrocytes (H). **I.** Heatmap depicting critical genes that define two astrocyte subtypes. **J.** clusterProfiler GO enrichment items of biological process for DEGs in P60 astrocytes. **K.** UMAP plot of all *Col23a1*+ tanycytes (top), and *Vcan*+ α-tanycytes (green) and *Col25a1*+ β-tanycytes (blue, bottom). **L.** Volcano plot depicting DEGs of P60 tanycytes. **M.** Violin plots showing significant downregulation of *Fgf14* in H3.3K4M Hom tanycytes. PMN, premammillary nucleus; VMH, ventromedial hypothalamus; LH, lateral hypothalamus; DMN, dorsomedial nucleus; Prethal, Prethalamus; ARC, Arcuate nucleus; ID&TT, intrahypothalamic diagonal & tuberomammillary terminal; SMN, supramammillary nucleus; PVN/SON, paraventricular nucleus/supraoptic nucleus; Ant ID, Anterior intrahypothalamic diagonal; SCN, suprachiasmatic nucleus; MMN, mammillary nucleus.

bioRxiv preprint doi: https://doi.org/10.1101/2025.05.02.651761; this version posted May 2, 2025. The copyright holder for this preprint (which was not certified by peer review) is the author/funder. This article is a US Government work. It is not subject to copyright under 17 USC 105 and is also made available for use under a CC0 license.

Figure S9

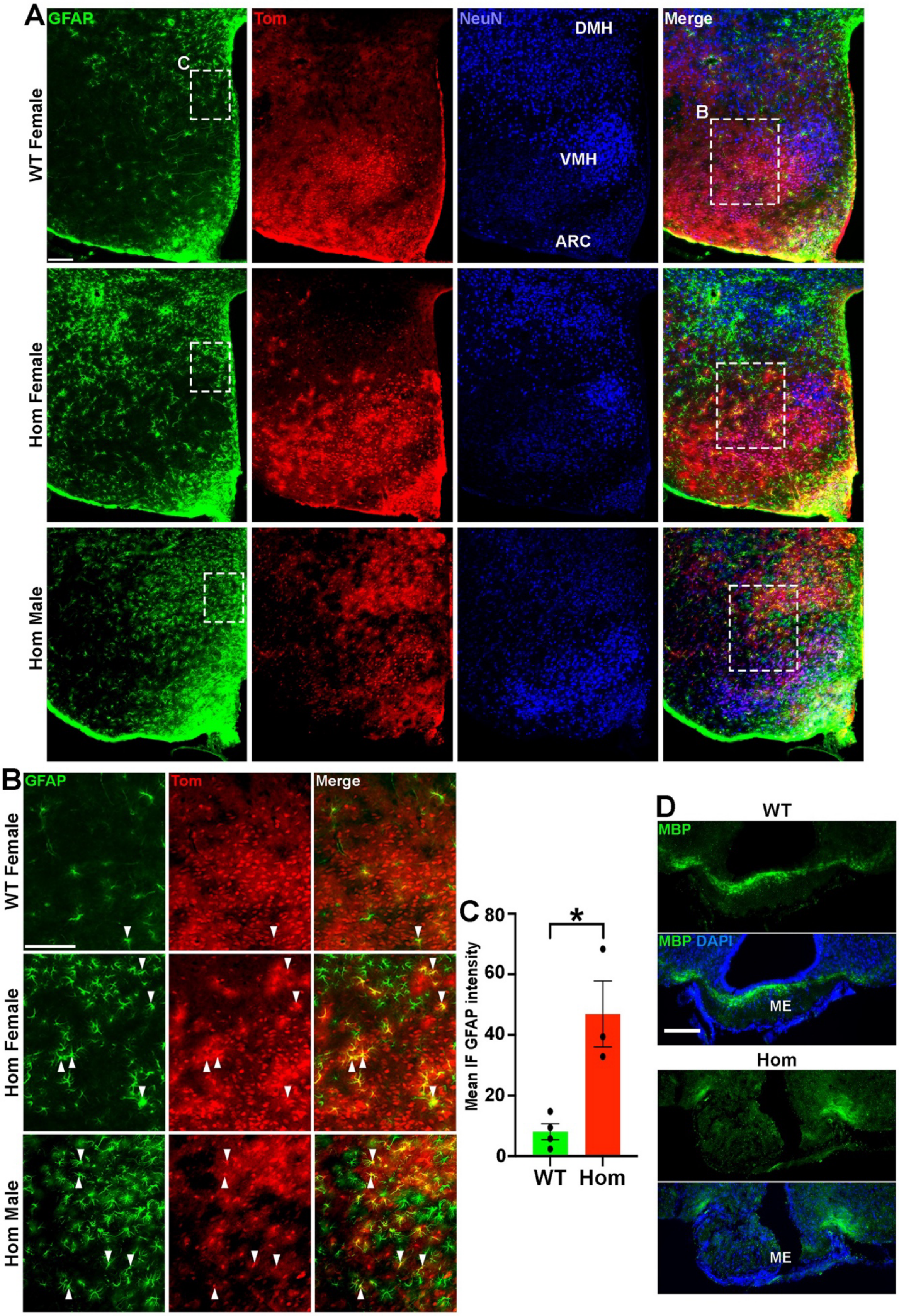

bioRxiv preprint doi: https://doi.org/10.1101/2025.05.02.651761; this version posted May 2, 2025. The copyright holder for this preprint (which was not certified by peer review) is the author/funder. This article is a US Government work. It is not subject to copyright under 17 USC 105 and is also made available for use under a CC0 license.

Figure S9. Dysregulation of glia cells in adult hypothalamus of H3.3K4M Hom mice. A-C. Representative images of adult hypothalamus from H3.3K4M WT and Hom male and female mice stained for GFAP (green), NeuN (blue), which Nkx2.1-linage cells expressing tdTomato. Scale bar = 100 μm. White rectangles in A indicate higher magnification regions (B), and regions used to measure immunofluorescence (IF) intensity of GFAP signal (C). **D.** Representative images of adult median eminence (ME) from H3.3K4M WT and Hom mice stained for MBP (green) and DAPI (blue). Scale bar = 100 μm. ARC, Arcuate nucleus; VMH, ventromedial hypothalamus; DMH, dorsomedial hypothalamus.
